# Prophylactic Use of Lipid Emulsion Therapy After Inadvertent Administration of 0.5% Ropivacaine for a Bier Block: A Case Report

**DOI:** 10.1155/cria/6254387

**Published:** 2025-12-22

**Authors:** Alexander Luong, Sirimas Lau, Elizabeth Johnson-Gray, Rayyan Bhutta, Austin Shaffer, Mitchell Hughes, Andrew Gable, Matthias Franzen

**Affiliations:** ^1^ Anesthesiology Residency Program, OhioHealth Doctors Hospital, Columbus, Ohio, USA; ^2^ Ohio University Heritage College of Osteopathic Medicine, Dublin, Ohio, USA, ohio.edu

**Keywords:** Bier block, carpal tunnel surgery, local anesthesia, local anesthetic toxicity, ropivacaine

## Abstract

The Bier block for intravenous regional anesthesia is generally well tolerated, and few complications have been reported. It provides a bloodless surgical field and adequate analgesia when performed correctly. A diluted short‐acting local anesthetic such as 0.5% lidocaine is typically used. Our case is a 41‐year‐old female who presented for endoscopic carpal tunnel release of her right wrist. An accidental injection of 30 mL of 0.5% ropivacaine was given instead of the normal 30 mL of 0.5% lidocaine, with a total tourniquet time of 46 min. Although the maximum safe dose of local anesthetic was not exceeded, lipid emulsion was administered out of an abundance of caution. No neurological or cardiovascular symptoms were reported, and the patient was discharged later that day.

## 1. Introduction

Intravenous regional anesthesia (IVRA), first described by Austin Bier in 1908 [1], is a popular anesthetic technique used for minor surgical procedures of the upper extremities, including carpal tunnel surgery. Advantages of the Bier block include its simplicity, reliability, and cost‐effectiveness. Often used in the ambulatory setting, the Bier block is limited to short procedures which are generally under 60 min [2].

Due to its rapid onset, short duration, and substantially reduced cardiotoxicity profile as compared with long‐acting local anesthetics, lidocaine is the preferred and recommended agent for IVRA [3]. In the light of their higher potency, as well as the higher risk of systemic toxicity, if any fraction reaches the circulation, long‐acting medications, like ropivacaine and bupivacaine, are deliberately avoided for Bier blocks. Local anesthetic systemic toxicity (LAST) is a known side effect of IVRA, although it is rare. This is particularly the case when an unintentional agent or an excessive dosage enters the circulation [4].

Local anesthetics produce anesthesia by blocking action potential conduction in the peripheral nerves. The local anesthetic binds to membrane‐bound sodium channels in the neuron, causing a reversible inhibition of the channel. An influx of sodium is required for neuron depolarization and subsequent propagation of impulses [5]. As the nerve loses the ability to depolarize and propagate impulses, the patient loses sensation in the area supplied by that particular nerve [5, 6].

While all amide local anesthetics have these characteristics, ropivacaine has slower dissociation kinetics and a higher sodium‐channel binding affinity than lidocaine. These characteristics make it more likely to cause cardiac depression and arrhythmias if administered systemically [7].

This case is presented to highlight the risks associated with inadvertent administration of a long‐acting anesthetic during IVRA and the clinical decision‐making that guided prophylactic lipid therapy.

## 2. Case Report

The patient was a 41‐year‐old healthy female, ASA II African American patient weighing 90 kg with a past medical history of acid reflux and hyperparathyroidism who presented for an endoscopic carpal tunnel release of her right wrist. Preoperative examination for this patient was unremarkable; cardiac and respiratory examinations were within normal limits. Physical examination was without any obvious deformities or lesions to the skin. Two preoperative intravenous catheters were placed without difficulty, one in each of the patient’s hands.

Upon arrival to the operating room, the patient was given 2 mg of midazolam, and a propofol infusion was started at 50 mcg/kg/mL. A Bier block was then successfully performed on the patient’s right arm.

A double‐cuffed pneumatic tourniquet was placed on the upper arm at standard inflation pressure (250–300 mmHg), and the right upper extremity was exsanguinated using an Esmarch bandage. In order to achieve total blockage, the proximal cuff was inflated. 30 mL of local anesthetic was administered through the distal IV catheter as the tourniquet was inflated. Tourniquet inflation started at the beginning of the operation and lasted 46 min in total, including 32 min during the procedure and 14 min during the lipid infusion; deflation did not occur until lipid therapy had concluded.

**Figure 1:**
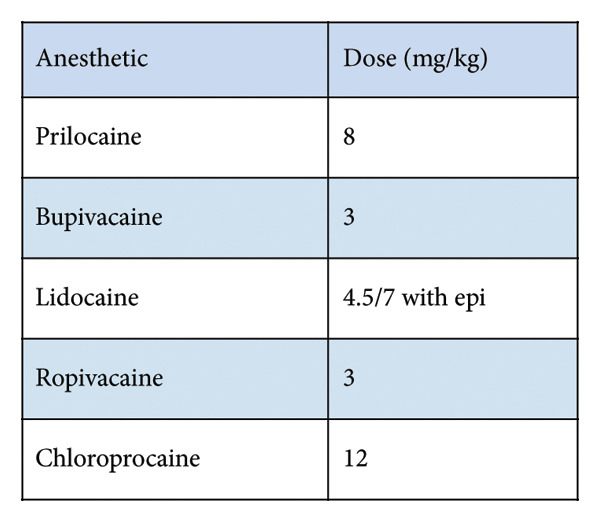
Local anesthetic toxicity.

**Figure 2:**
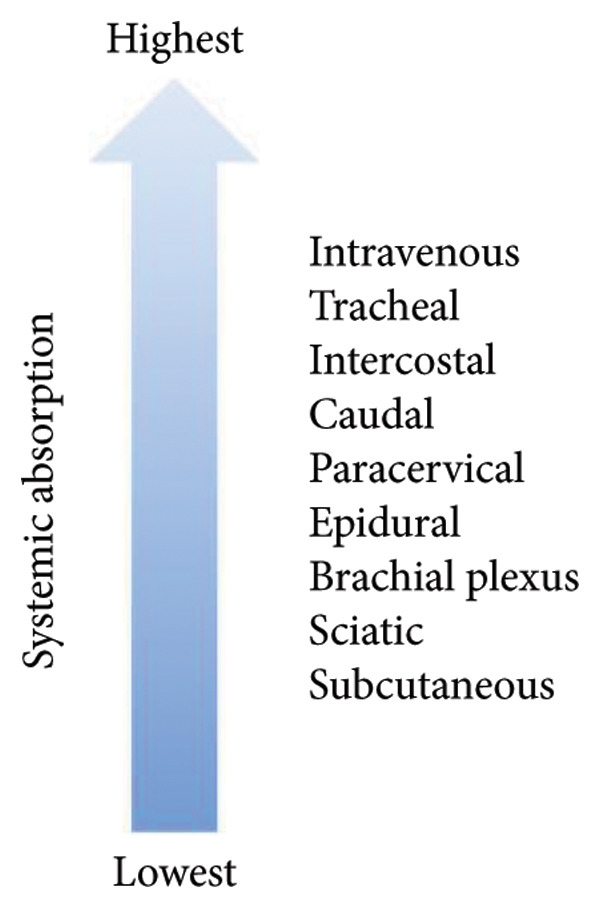
Local absorption.

About 30 min into the procedure, while the tourniquet was still inflated, it was noted that the certified registered nurse anesthetist (CRNA) had inadvertently performed the Bier block with 30 mL of 0.5% ropivacaine instead of the intended 0.5% lidocaine. After informing the surgeon, a decision was made to discontinue the propofol infusion and initiate lipid emulsion. The surgeon was notified immediately, and as the patient was hemodynamically stable and surgical closure was already underway, he elected to complete the procedure while lipid therapy was initiated. Although the patient had not exceeded the maximum dose of ropivacaine, the dose was given IV, and this route has the highest rate of absorption (Figures 1 and 2). A bolus dose of 1.5 mL/kg, followed by an infusion of 0.25 mL/kg/min of Intralipid, was administered intravenously. The patient was then continuously monitored throughout the procedure for neurological and cardiovascular signs of LAST, and the patient remained asymptomatic throughout.

After the completion of the infusion, the patient was reevaluated in the postanesthesia care unit (PACU). Her neurologic examination was intact, and she did not endorse any skin irritation, pain at the injection site, or neurological issues. She was informed of the accidental dose of ropivacaine given instead of lidocaine and that she was treated with Intralipid therapy. After being discharged from the hospital, she was given instructions to keep a watch out for any delayed signs of systemic illness, such as tinnitus, perioral numbness, dizziness, palpitations, confusion, or seizures. She was instructed to call the hospital’s 24‐h anesthetic service and to return to the emergency room if she developed any neurological or cardiovascular concerns. The surgical team arranged an early postoperative follow‐up within 4 days of the procedure. She was hemodynamically stable and discharged home later that day. At her early follow‐up visit, she remained asymptomatic, and at her one‐month postoperative appointment, she continued to report no neurological or cardiovascular issues.

## 3. Discussion

Common practice for the Bier block for the upper extremities includes inserting a distal intravenous catheter in the hand, exsanguinating the extremity with an Esmarch bandage, and then applying a double‐cuffed pneumatic tourniquet on that same extremity to achieve exsanguination of that extremity [2]. Once the double‐cuffed tourniquet is inflated, local anesthetic can then be administered: typically 20–30 mL of 0.5% lidocaine, with onset of the block within 5 min. The tourniquet is inflated for a minimum of 15–20 min to prevent systemic circulation of the local anesthetic.

The regular procedure was followed in this instance; however, ropivacaine was mistaken for lidocaine during the medication‐administration process. The route of delivery made this error clinically significant because ropivacaine is not advised for IVRA due to its higher cardiotoxic potential [7]. Multiple case reports have documented severe LAST resulting from inadvertent intravascular administration of long‐acting local anesthetics during extremity blocks, with several instances requiring urgent lipid rescue for cardiovascular support [8–10].

LAST is a life‐threatening complication that can occur after the administration of regional anesthesia. The underlying mechanism is believed to be multifactorial, with symptoms affecting the central nervous system (CNS) and cardiovascular system (CVS). CNS symptoms generally present before CVS symptoms, the most common including tinnitus, perioral numbness, metallic taste in the mouth, or dizziness. More serious CNS symptoms include seizures or coma. CVS symptoms may include myocardial depression or cardiac arrest. Thanks to the use of ultrasound and limitation of drug dose, the incidence of LAST is estimated to be 0.03% [11].

Notably, once the error was discovered in this case, our patient presented no signs of neurological or cardiovascular problems. However, systemic exposure could happen immediately upon cuff removal due to the fact that the medication was delivered intravenously while the tourniquet was inflated. Despite the lack of clinical symptoms, this established a high‐risk window.

Although lipid rescue is traditionally initiated after symptoms appear, several published cases involving accidental intravascular injection of long‐acting agents have supported early or prophylactic administration to prevent rapid cardiovascular collapse [11]. This rationale guided our decision to begin lipid therapy before tourniquet deflation. To our knowledge, there are no previously reported cases describing prophylactic lipid therapy following accidental intravenous ropivacaine administration specifically during IVRA, highlighting the uniqueness and clinical relevance of this case.

The definitive treatment of LAST includes seizure management, advanced cardiac life support (ACLS), and early initiation of lipid emulsion therapy [12]. Epinephrine doses should be reduced to 1 mcg/kg if used, instead of the typical 1 mg per dose in ACLS protocol and drugs such as beta‐blockers, calcium channel blockers, and other local anesthetics should be avoided [12]. The mechanism of lipid emulsion therapy is multimodal; the lipid has both direct cardiotonic effects and acts as a scavenger of lipophilic moieties [12, 13]. The scavenging effect allows the lipid emulsion to act as a “shuttle,” facilitating the uptake of local anesthetics into the liver, thereby accelerating redistribution from the plasma [14]. Dosing for 20% Intralipid therapy is a 1.5 mL/kg initial bolus followed by an infusion at 0.25 mL/kg/min. The bolus can be repeated, and the infusion can be doubled in cases of hemodynamic instability.

The administration of lipid emulsion rescue is indicated as one of the three pillars of LAST treatment, along with ACLS and seizure management [15]. Intralipid has also been indicated in recent years as a therapeutic agent in the reversal of other drug overdoses, such as certain antipsychotics, antidepressants, antiarrhythmics, and calcium channel blockers. In the case of our patient, upon recognition of the mistake, Intralipid prophylaxis was started immediately to combat a possibility of LAST from ropivacaine, resulting in no signs of cardiac or neurological toxicity.

Reports of LAST associated with Bier blocks typically include lidocaine and are more frequently linked to early cuff release rather than mistakes in drug selection. This case is a valuable contribution to the existing literature considering that there are currently no documented examples that discuss prophylactic lipid therapy for unintentional intravenous ropivacaine during IVRA. Our patient remained asymptomatic throughout, which demonstrates the potential benefit of early detection and preventive lipid therapy in high‐risk IVRA errors, in contrast with previously reported reports of LAST following upper‐extremity blocks where patients experienced seizures or cardiovascular collapse prior to receiving lipid rescue [8].

## 4. Conclusion

This case highlights the importance of careful medication administration and monitoring during surgical procedures. Despite the inadvertent administration of 0.5% ropivacaine instead of the intended 0.5% lidocaine during the Bier block, the patient did not exhibit any immediate neurological or cardiovascular symptoms. However, given the potential risks associated with the unintentional dosing of ropivacaine, the decision to initiate lipid emulsion therapy was made to mitigate the risk of toxicity.

## Consent

Informed consent was obtained from the patient prior to manuscript submission.

## Conflicts of Interest

The authors declare no conflicts of interest.

## Funding

No external source of funds was used for the publication of this case report.

## Data Availability

Data sharing is not applicable to this article as no datasets were generated or analyzed during the current study.
